# Medical School Policies on Faculty Participation in Industry-Sponsored Speakers’ Bureaus

**DOI:** 10.1001/jamanetworkopen.2025.32325

**Published:** 2025-09-17

**Authors:** Jack R. Wieberdink, Constance P. Chen, Marcus N. Milani, Brandon E. Semke, Jonathan D. Alpern, David J. Satin

**Affiliations:** 1 University of Minnesota Medical School, Minneapolis; 2 Division of Infectious Diseases, Department of Medicine, Minneapolis VA Medical Center, Minneapolis, Minnesota; 3 Department of Medicine, University of Minnesota, Minneapolis; 4 Department of Family Medicine, University of Minnesota, Minneapolis; 5 Center for Bioethics, University of Minnesota, Minneapolis

## Abstract

This cross-sectional study analyzes US allopathic medical school conflict of interest policies on faculty participation in industry-sponsored speakers’ bureaus.

## Introduction

Pharmaceutical companies commonly recruit and pay clinicians, often key opinion leaders in their field, to give educational presentations to other clinicians to promote company products.^1^ Industry-sponsored speakers’ bureaus (ISSBs) represent a controversial subset of these relationships, characterized by faculty operating as paid spokespersons while relinquishing substantial control of the educational content to the pharmaceutical company.^2^ On November 16, 2020, the US Office of Inspector General issued a special fraud alert of cases alleging that large speaker payments were being made to high-prescribing physicians on speakers’ bureaus, violating the antikickback statute. Because of the risk of undue influence from faculty engaging with industry while providing medical care and education purported to be objective, the Institute of Medicine recommended that academic medical centers prohibit faculty from delivering industry-controlled educational presentations.^2^ Similarly, the American Medical Student Association (AMSA) developed a model policy forbidding faculty from participating in promotional speakers’ bureaus.^3^ It has been more than a decade since the AMSA analyzed conflict of interest (COI) policies of US medical schools.^3,4^ In this study, we provide a contemporary analysis of US medical school policies on faculty participation in ISSBs.

## Methods

In this cross-sectional study, we collected COI policies pertaining to ISSBs from US allopathic medical school websites and through email requests between August 26, 2024, and March 10, 2025. This study was determined to be nonhuman participants research by the Minneapolis Veterans Affairs Research and Development Committee. This report followed the STROBE reporting guideline.

Policies regarding ISSBs were coded as explicitly prohibited, prohibited with exceptions or allowed with conditions, discouraged without conditions, explicitly allowed, or not addressed (further details provided in the eMethods in Supplement 1). Each policy was independently coded by 2 authors (J.R.W. and C.P.C.) with 92% (119 of 129 policies) agreement. Two additional authors (M.N.M. and B.E.S.) independently coded the 10 policies with disagreement, resolving 8 through unanimous agreement. The 2 senior authors (J.D.A. and D.J.S.) independently coded the final 2 policies, and agreement was achieved as a team through consensus. Descriptive statistics were performed using Excel, version 2108 (Microsoft Corp).

## Results

We obtained 129 COI policies from 159 US allopathic medical schools (81%). Forty-nine COI policies (38%) explicitly prohibited ISSBs, 25 (19%) prohibited ISSBs with exceptions or permitted ISSBs with conditions, 11 (9%) discouraged ISSBs without conditions, 17 (13%) explicitly permitted ISSBs, and 27 (21%) did not address ISSBs (Figure). Policy exceptions and conditions are typically afforded to faculty who believe that they have creative content control.

**Figure.**
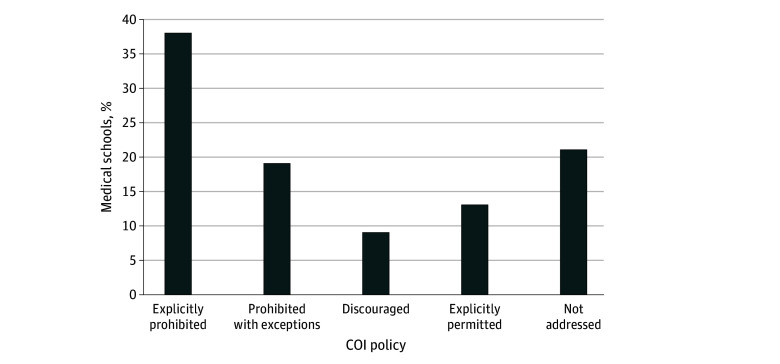
US Allopathic Medical School Policies on Faculty Participation in Industry-Sponsored Speakers’ Bureaus COI indicates conflict of interest.

## Discussion

Despite increased awareness that faculty participation in ISSBs threatens academic independence,^5,6^ this cross-sectional study found that only 38% of US allopathic medical schools explicitly prohibit this practice. That at least 50% did so on the 2014 AMSA Scorecard^3^ suggests that medical schools’ ISSB policies have become less strict over the past decade. We found that the language used to define ISSBs, and to determine their permissibility, varied and was often subject to interpretation. Although many policies use language to dissuade faculty from participating in ISSBs, stopping short of explicitly prohibiting ISSBs essentially permits these industry relationships. Allowing faculty with an ISSB COI to determine whether they believe that they have creative control may also permit these relationships. Study limitations include that we only analyzed allopathic medical schools, and policies were subject to interpretation, which may have led to misclassification.

Industry-sponsored speakers’ bureaus are among the highest-risk COIs in health care. To ensure that faculty and clinical practice remain independent from industry influence, US medical schools should adopt the best practice of explicitly prohibiting faculty from participating in ISSBs.^3^ Further research is needed to determine the impact of COI policies on faculty behavior.

## Notes

Supplement 1.
eMethods.

eReference.

Supplement 2.
Data Sharing Statement
