# Supplementary material for: Medical School Policies on Faculty Participation in Industry-Sponsored Speakers’ Bureaus
Source: JAMA Netw Open. 2025 Sep 17;8(9):e2532325. doi: 10.1001/jamanetworkopen.2025.32325 (PMC12444573; doi:10.1001/jamanetworkopen.2025.32325)
Supplement: Supplement 1. — eMethods. eReference. [file jamanetwopen-e2532325-s001.pdf]

Supplemental Online Content

Wieberdink JR, Chen CP, Milani MN, Semke BE, Alpern JD, Satin DJ. Medical school policies on faculty participation in industry-sponsored speakers' bureaus. *JAMA Netw Open*. 2025;8(9):e2532325. doi:10.1001/jamanetworkopen.2025.32325

eMethods

eReference

This supplemental material has been provided by the authors to give readers additional information about their work.

© 2025 Wieberdink JR et al. *JAMA Network Open*.

eMethods

We obtained a list of US allopathic medical schools from the Liaison Committee on Medical Education.[1] For each medical school, we emailed a school administrator and searched the internet for a publicly available COI policy that addressed faculty participation in ISSBs. To obtain COI policies, we searched for medical school policies pertaining broadly to COIs as well as more specific policies that addressed faculty relationships with industry including Frequently Asked Question documents. We used the policy that most closely addressed ISSBs. We analyzed both the emailed policy and the publicly available policy, if available, for each school. If the policies differed, we used the policy that most closely addressed ISSBs. If both policies addressed ISSBs, we used the more restrictive policy.

We analyzed policies from the perspective of whether a faculty member could reasonably find justification in the policy to participate in an ISSB. We initially coded the policies as follows: ISSBs explicitly prohibited, prohibited with exceptions/allowed with conditions, discouraged without conditions, explicitly allowed, or ISSBs "not mentioned". Policies that prohibited faculty from participating in ISSBs and did not include any qualifying statements or exceptions to the rule were considered to "explicitly prohibit" ISSBs. The category "prohibited with exception/allowed with conditions" was broad in scope and incorporated policies indicating that ISSBs a) were prohibited but with exceptions under which it could be allowed, b) allowed but under certain conditions, and c) are allowed but that the school ultimately made the final decision about whether it would be allowed.

To account for policies that did not explicitly mention ISSBs but used verbiage describing them (i.e. "company controls educational content"), we re-analyzed all the "ISSBs not mentioned" policies and categorized these as "ISSBs not addressed" if the policies did not mention or describe ISSBs at all. By contrast, if the policy originally coded as "ISSBs not mentioned" did use language that describe ISSBs, we re-coded these policies as: explicitly allowed, discouraged, prohibited with exceptions, or explicitly prohibited. The final categories were: ISSBs explicitly prohibited, prohibited with exceptions/allowed with conditions, discouraged without conditions, explicitly allowed, or ISSBs not addressed.

© 2025 Wieberdink JR et al. *JAMA Network Open*.

eReference

1. LCME | Liaison Committee on Medical Education. Accessed March 5, 2025. https://lcme.org/

© 2025 Wieberdink JR et al. *JAMA Network Open*.
