# Supplementary material for: Medical School Policies on Faculty Participation in Industry-Sponsored Speakers’ Bureaus
Source: JAMA Netw Open. 2025 Sep 17;8(9):e2532325. doi: 10.1001/jamanetworkopen.2025.32325 (PMC12444573; doi:10.1001/jamanetworkopen.2025.32325)
Supplement: Supplement 2. — Data Sharing Statement [file jamanetwopen-e2532325-s002.pdf]

Data Sharing Statement

Wieberdink. Medical School Policies on Faculty Participation in Industry-Sponsored Speakers' Bureaus. *JAMA Netw Open*. Published September 17, 2025.
doi:10.1001/jamanetworkopen.2025.32325

Data
Data available: Yes
Data types: Participant data with identifiers
How to access data: Happy to share data with reviewers/journal upon request.
When available: With publication

Supporting Documents
Document types: None

Additional Information
Who can access the data: Upon request
Types of analyses: NA
Mechanisms of data availability: NA
